# Causal association of sarcopenia-related traits with brain cortical structure: a bidirectional Mendelian randomization study

**DOI:** 10.1007/s40520-025-02977-x

**Published:** 2025-02-27

**Authors:** Yuxuan Zhan, Zhiyun Zhang, Siyi Lin, Bang Du, Kai Zhang, Jian Wu, Hongxia Xu

**Affiliations:** 1 School of Public Health, Institute of Wenzhou, Zhejiang University, Hangzhou, 310058 China; 2 Department of Infectious Diseases, The Second Affiliated Hospital, Zhejiang University School of Medicine, Hangzhou, 310009 China; 3 WeDoctor Cloud and Liangzhu Laboratory, Hangzhou, 310000 China; 4 Zhejiang Key Laboratory of Medical Imaging Artificial Intelligence, Zhejiang University, Hangzhou, 310000 China

**Keywords:** Sarcopenia, Brain structure, Bidirectional Mendelian randomization, Muscle-brain axis

## Abstract

**Background:**

Patients with sarcopenia often experience cognitive decline, affecting cortical structures, but the causal link remains unclear. We used bidirectional Mendelian randomization (MR) to explore the relationship between sarcopenia-related traits and cortical structure.

**Methods:**

We selected genetic variables from genome-wide association study data. Three different MR methods were used: inverse-variance weighted analysis, MR-Egger regression, and the weighted median test. For significant estimates, we further conducted Cochran’s Q test, MR-Egger intercept test, leave-one-out analyses, and MR-PRESSO to assess heterogeneity.

**Results:**

In forward MR analysis, appendicular lean mass (ALM) decreased the thickness (TH) of lateral occipital gyrus and increased the TH of pars opercularis gyrus (β = -0.0079 mm, 95% CI: -0.0117 mm to -0.0041 mm, *P* < 0.0001; β = 0.0080 mm, 95% CI: 0.0042 mm to 0.0117 mm, *P* < 0.0001). In reverse MR analysis, a significant negative correlation was found between the TH of bankssts and ALM, while positive correlations were observed between the TH of frontal pole, rostral anterior cingulate, temporal pole, and ALM. The TH of temporal pole was positively correlated with right hand grip strength (HGS-R) (β = 0.1596 mm, 95% CI: 0.1349 mm to 0.1843 mm, *P* < 0.0001), and the TH of pars triangularis was positively correlated with left-hand grip strength (HGS-L) (β = 0.3251 mm, 95% CI: 0.2339 mm to 0.4163 mm, *P* < 0.0001).

**Conclusions:**

Sarcopenia-related traits and cortical structure have bidirectional effects, supporting the muscle-brain axis theory. This links sarcopenia to neurocognitive diseases and provides new strategies for the prevention and intervention of both sarcopenia and cognitive decline.

**Supplementary Information:**

The online version contains supplementary material available at 10.1007/s40520-025-02977-x.

## Introduction

Sarcopenia, a condition commonly found among the elderly, is characterized by progressive and generalized deterioration of muscle mass and function [1]. The morbidity rate associated with sarcopenia falls within the range of 10–27% [2]. Numerous observational studies have revealed a higher occurrence of cognitive impairment among individuals with sarcopenia compared to those without sarcopenia [3, 4]. Moreover, the concurrent presence of sarcopenia and cognitive decline is becoming increasingly common [5–7]and can be accompanied by various neurological disorders [8], such as dementia [9], stroke [10], and Parkinson’s disease [11]. These neurological conditions are often accompanied by changes in brain structure and function [12–14], which may suggest the existence and regulation of the muscle-brain axis [15, 16]. However, since there is no standardized cut-off point for sarcopenia diagnostics, the association between muscle mass and cognitive impairment has not reached a consensus [17], and the underlying factors driving this association are not yet fully understood. Therefore, investigating the relationship between muscle and brain functions during cognitive impairment may provide new insights and explanations for this phenomenon.

Some cohort studies have demonstrated the coexistence of brain atrophy and muscle atrophy [3], providing evidence for a relationship between sarcopenia and brain structure [18]. However, in certain study populations, no significant correlation was discovered between muscle loss and brain structure [19], or it was suggested that the correlation between white and gray matter quality and muscle mass or strength was weak [20]. So further investigation is necessary due to the inconsistencies found in the current body of research. Previous studies also have limitations resulting from the unequal distribution of socio-demographic samples and the impact of confounding factors like smoking and alcohol consumption. Therefore, additional research is needed to address these gaps and provide more comprehensive insights into the topic.

Mendelian randomization (MR) avoids confounding variables and reverse causality by using genetic variation as an instrumental variable to study causal effects associated with exposure and outcome. Several studies have been conducted to explore the relationship between sarcopenia and neurological disorders through MR analysis [21, 22]. However, no MR analysis has been conducted to discover the relationship between sarcopenia-related traits and brain cortex structure alternation to our knowledge. We adopted bidirectional two-sample MR analysis, which uses two independent sets of summary statistics from genome-wide association studies (GWASs). This method overcomes the effects of confounding by screening for genomic variants and provides more reliable results.

Our study seeks to elucidate the bidirectional relationship between sarcopenia-related traits and brain cortex structure using human genetic data. The brain cortex structure is measured by cortical surface area (SA) and thickness (TH), as detected using Magnetic Resonance Imaging (MRI). We adopted three sets of measurements to evaluate sarcopenia: appendicular lean mass [23], body fat percentage [24], and grip strength [25].

## Materials and methods

### Study design

**Fig. 1:**
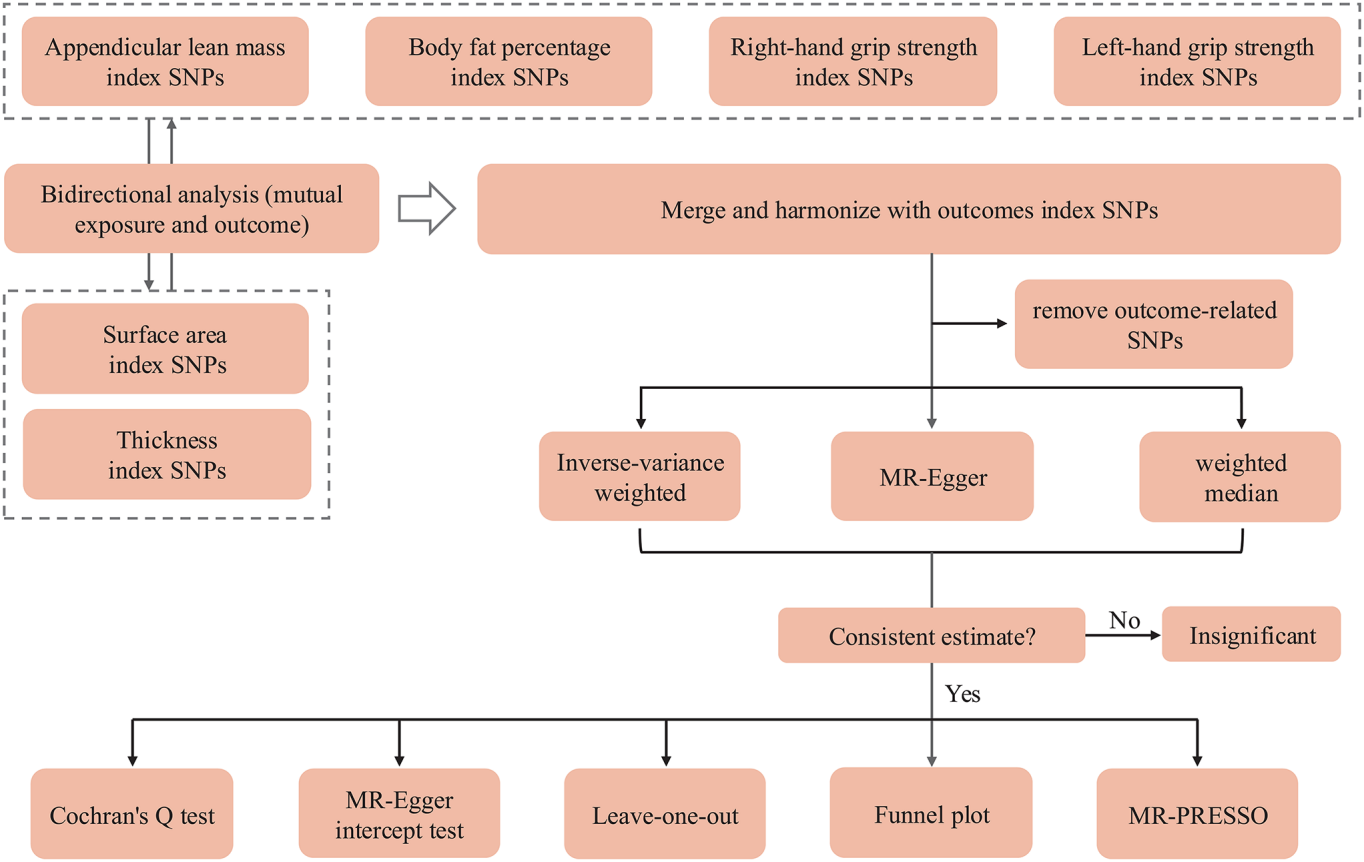
A flow chart of Mendelian randomization

This study was a bidirectional two-sample Mendelian randomization study reported according to STROBE-MR guidelines [26]. All SNP information in the study was publicly available from published summary statistics of the GWASs consortia website, Open GWAS (https://gwas.mrcieu.ac.uk/), and ENIGMA (http://enigma.ini.usc.edu/). Table 1 lists the concrete consortium and the GWAS ID of each phenotype. To avoid bias from ethnic background and sample overlapping, the individuals in our study were of European descent, and the cohorts of exposures and outcomes were relatively independent. In the present study, we examined the causal effects of four sarcopenia-related traits on brain cortical structure. Figure 1 displays the flow chart of our study design.

**Table 1:** Details of studies included in Mendelian randomization analyses

Phenotype	Consortium	Participants	GWAS ID	Sample size	Years of publication
Surface area	ENIGMA	European	NA	51,665	2020
Thickness	ENIGMA	European	NA	51,665	2020
Appendicular lean mass	UK Biobank	European	ebi-a-GCST90000025	450,243	2020
Body fat percentage	UK Biobank	European	ukb-b-8909	454,633	2018
Right-hand grip strength	UK Biobank	European	ukb-b-10,215	461,089	2018
Left-hand grip strength	UK Biobank	European	ukb-b-7478	461,026	2018

### GWAS summary data for sarcopenia-related traits

Sarcopenia symptoms typically manifest as a combination of clinical signs. According to the updated consensus from the European Working Group on Sarcopenia in Older People 2 (EWGSOP2), sarcopenia is diagnosed based on three criteria: low muscle strength, low muscle mass or quality, and low physical performance [27]. We selected key characteristics related to sarcopenia as our research factors.

Appendicular lean mass (ALM) is a genetic trait associated with lean muscle mass and sarcopenia, and can represent muscle quality [28, 29]. ALM was qualified by the sum of fat-free mass calculated by bioelectrical impedance analysis (BIA) using the TanitaBC418MA body composition analyzer [30], and available in 450,243 UK Biobank participants. Body fat percentage (BFP) can reflect physical performance [31]. GWAS data of BFP were measured following the same protocol as ALM among 454,633 individuals from the UK Biobank. Hand grip strength (kg) validly represents muscle strength, which was measured using a Jamar J00105 hydraulic hand dynamometer adjusted for hand size [30]. Right-hand grip strength (HGS-R) and left-hand grip strength (HGS-L) were used to represent muscle strength [32]. We separately analyzed the causal effects of HGS-R with 461,089 UK Biobank cohort participants and HGS-L with 461,026 UK Biobank cohort participants on brain cortical structure.

### GWAS summary data for brain cortical structure

Genetic variant association estimates with the brain cortical structure were selected from a GWAS meta-analysis of brain MRI data processed by the ENIGMA Consortium [33]. The brain cortical SA and TH were analyzed in 51,665 individuals, primarily (~ 94%) of European descent, across 60 cohorts worldwide. The detailed cohorts were listed in Supplementary File 1 Table S1. 34 brain functional specializations were defined by the commonly used Desikan-Killiany atlas based on the gyrus and were averaged between both hemispheres [33].

### Genetic instruments selection

The selection of instrumental variables (IVs) is the core process of the MR analysis, which determines the accuracy and reliability of the causal inferences. We sorted the SNPs as IVs under three fundamental assumptions: (1) the relevance assumption: the SNPs are associated with the exposure; (2) the independence assumption: the SNPs are independent of the other confounding factors of outcome; (3) the exclusion restriction assumption: the SNPs are independent of the outcome and can only affect the outcome through the exposure [34].

To meet the assumptions, the following steps were conducted. Firstly, the SNPs with a GWAS-correlated *P*-value higher than 5 × 10^− 8^ were excluded. To obtain IVs with no linkage disequilibrium (LD), we clumped the SNPs with the standard of r^2^ < 0.01 and kb = 10,000. Then, we calculated the F-statistics with each SNPs and discarded the SNPs with F-statistics less than 10. For each SNP, F statistics > 10 indicated a strong relationship [35]and were calculated using the formula:$\:F=\frac{{R}^{2}\times\:\left(N-2\right)}{1-{R}^{2}}$. And R^2^ was calculated using the formula:$\:{R}^{2}=\frac{{\beta\:}^{2}}{{\beta\:}^{2}+S{E}^{2}\times\:N}$. In the inverse MR analysis for causal estimation of brain structures on sarcopenia-related traits, we set the genome-wide significance threshold at *P* < 5 × 10^−6^. We relaxed the statistical threshold for selecting instrumental variables (IVs) since very few or no SNPs were identified when a threshold of *P* < 5 × 10^−8^ was used [36].

We also checked in PhenoScanner [37] (http://www.phenoscanner.medschl.cam.ac.uk/), a platform with comprehensive information on the association of genotype and phenotype, to see whether these SNPs were associated with the potential risk factors, including neuropsychiatric disease, hypertension, hyperlipemia, alcohol, stroke, obesity, smoking, vitamin D, educational attainment, and anxiety, then remove SNPs associated with any of these potential confounders at genome-wide significance.

### Mendelian randomization analysis

Bidirectional MR analysis was conducted to determine the mutual associations between sarcopenia-related traits (ALM, BFP, HGS-R, HGS-L) and the cortical structures and functional regions of the whole brain. To increase the credibility of the results, we used three different MR methods: inverse-variance weighted (IVW) analysis, MR-Egger regression, and weighted median test. The IVW is the most common analysis method based on the assumption that all IVs are valid [38]and is computed as the SNP-to-outcome association divided by the SNP-to-exposure association [38]. In the absence of directional pleiotropy between IVs, the bias of IVW can be ignored and the result is considered the most reliable indicator of causal relationship. The MR-Egger method and weighted median test are used to improve the accuracy of IVW estimation. Weighted median allows that < 50% of the IVs are invalid, and MR-Egger allows the presence of imbalanced horizontal pleiotropy and provides estimates after adjusting for pleiotropic effects [38]. The estimates were considered significant, only if the *P*-value of IVW was less than 0.05 and the estimates of β directions of IVW and weighted median test were consistent. For significant estimates, we further used Cochran’s Q test to assess heterogeneity, and *P*_Q_ < 0.05 was considered significant in heterogeneity. The MR-Egger intercept test, leave-one-out analyses and the MR-PRESSO test were also applied to evaluate the horizontal pleiotropy. If the *P*-vale of intercept < 0.05, there is horizontal pleiotropy, and the result should be discarded. The fixed effects model was used if the heterogeneity was negligible. Otherwise, the IVW with random effects approach was acceptable.

### Statistics

All MR analyses were conducted using R software (version 4.2.0) with the packages “TwoSampleMR” (version 0.5.7). For estimates at region-level, a Bonferroni-corrected *P-*value was set as 1.74 × 10^− 4^(0.05/288[36 × 4 × 2]; 36 represents the number of brain functional regions, 4 represents the number of sleep traits, and 2 represents both forward and reverse MR analyses), while the *P* < 0.05 suggested normally significant.

## Results

### Overview of the study

We conducted a bidirectional two-sample MR analysis to investigate the relationships between all sarcopenia-related traits and brain regions. The study flowchart is shown in Fig. 1. We identified several significant or nominally significant brain structural regions influenced by sarcopenia-related traits (Table 2), as well as several sarcopenia-related traits influenced by brain outcome regions (Table 3). The results of the sensitivity analysis are presented in Tables 4 and 5. Scatter plots and leave-one-out plots of significant estimates from both forward and reverse Mendelian randomization analyses can be found in Supplementary File 1, Figure S1.

**Table 2:** IVW estimates for sarcopenia-related traits on brain structure (Forward Mendelian randomization Analysis)

	Exposures	Outcomes	SNPs	β(95%CI)	SE	*P*-value
Significant estimates***	ALM	TH of lateral occipital	278	-0.0079(-0.0117,-0.0041)	0.0019	< 0.0001
		TH of pars opercularis	278	0.0080(0.0042,0.0117)	0.0019	< 0.0001
Nominal significant Estimates*	ALM	SA of entorhinal	460	2.8765(0.1690,5.5839)	1.3814	0.0373
		SA of inferior parietal	461	19.3798(1.4771,37.2824)	9.1340	0.0339
		SA of insula	461	10.6153(2.5422,18.6884)	4.1189	0.0100
		SA of lateral occipital	462	-29.7352(-46.9629,-12.5075)	8.7896	0.0007
		SA of lateral orbitofrontal	463	8.8693(0.3090,17.4296)	4.3675	0.0423
		SA of lingual	463	-14.7574(-28.2231,-1.2917)	6.8703	0.0317
		SA of pars opercularis	462	9.6748(2.2221,17.1275)	3.8024	0.0109
		SA of rostral middle frontal	462	-19.8897(-37.5771,-2.2022)	9.0242	0.0275
		TH of caudal middle frontal	463	0.0051(0.0011,0.0091)	0.0021	0.0125
		TH of cuneus	460	-0.0058(-0.0104,-0.0011)	0.0024	0.0146
		TH of entorhinal	463	-0.0167(-0.0301,-0.0033)	0.0068	0.0144
		TH of inferior parietal	463	-0.0051(-0.0083,-0.0019)	0.0016	0.0018
		TH of inferior temporal	463	-0.0079(-0.0125,-0.0034)	0.0023	0.0007
		TH of lateral orbito frontal	278	-0.0053(-0.0105,-0.0002)	0.0026	0.0427
		TH of rostral middle frontal	278	0.0050(0.0016,0.0084)	0.0017	0.0037
		TH of superior temporal	278	0.0047(0.0001,0.0092)	0.0023	0.0432
	BFP	TH of superior parietal	115	0.0136(0.0066,0.0207)	0.0036	0.0002
		SA of lateral occipital	463	46.8558(13.0451,80.6664)	17.2503	0.0066
		SA of pars opercularis	463	16.8636(0.9923,32.7349)	8.0976	0.0373
		SA of precentral	461	-34.0708(-64.4949,-3.6466)	15.5225	0.0282
		TH of caudal middle frontal	278	0.0090(0.0008,0.0172)	0.0042	0.0318
		TH of fusiform	127	-0.0122(-0.0214,-0.0030)	0.0047	0.0092
		TH of inferior parietal	127	0.0097(0.0035,0.0159)	0.0032	0.0022
		TH of middle temporal	127	-0.0093(-0.0182,-0.0003)	0.0046	0.0431
		TH of precuneus	115	0.0090(0.0021,0.0159)	0.0035	0.0109
	HGS-R	Global SA	462	5048.6960(2123.3746,7974.0174)	1492.5109	0.0007
		TH of insula	463	0.0272(0.0061,0.0483)	0.0108	0.0116
		TH of superior parietal	461	-0.0164(-0.0301,-0.0027)	0.0070	0.0186
		TH of superior temporal	462	0.0294(0.0133,0.0455)	0.0082	0.0004
	HGS-L	SA of superior temporal	463	49.8291(11.6132,88.0450)	19.4979	0.0106
		SA of temporal pole	460	9.2389(2.4248,16.0530)	3.4766	0.0079
		TH of bankssts	463	0.0210(0.0025,0.0396)	0.0095	0.0263
		TH of superior temporal	460	0.0182(0.0004,0.0360)	0.0091	0.0445

***Significant estimate is defined as IVW-derived *P* < 1.74*10^− 4^, *nominal significant estimate is defined as IVW-derived *P* < 0.05

**Table 3:** IVW estimates for brain structure on sarcopenia-related traits (Inverse Mendelian randomization Analysis)

	Exposures	Outcomes	SNPs	β(95%CI)	SE	*P*-value
**Significant**	TH of bankssts	ALM	12	-0.434(-0.6514,-0.2165)	0.1109	< 0.0001
estimates***	TH of frontal pole	ALM	40	0.2981(0.2412,0.355)	0.029	< 0.0001
	TH of rostral anterior cingulate	ALM	211	0.2672(0.2324,0.302)	0.0178	< 0.0001
	TH of temporal pole	ALM	12	0.3778(0.2478,0.5078)	0.0663	< 0.0001
	TH of temporal pole	HGS-R	137	0.1596(0.1349,0.1843)	0.0126	< 0.0001
	TH of pars triangularis	HGS-L	81	0.3251(0.2339,0.4163)	0.0465	< 0.0001
**Nominal significant**	TH of cuneus	ALM	18	0.4095(0.1957,0.6233)	0.1091	0.0002
Estimates*	TH of fusiform	ALM	23	0.2642(0.0554,0.473)	0.1065	0.0131
	TH of inferior parietal	ALM	6	-1.1768(-1.8321,-0.5214)	0.3343	0.0004
	SA of middle temporal	ALM	45	0.0001(0,0.0001)	< 0.0001	0.0163
	TH of pars triangularis	BFP	81	0.1503(0.062,0.2387)	0.0451	0.0009
	TH of pars orbitalis	HGS-R	137	-0.0848(-0.129,-0.0405)	0.0226	0.0002

***Significant estimate is defined as IVW-derived *P* < 1.74*10^− 4^, *nominal significant estimate is defined as IVW-derived *P* < 0.05

**Table 4:** Heterogeneity and Pleiotropy tests of the causal effects of sarcopenia-related traits on brain structure (Forward Mendelian randomization Analysis)

	Exposures	Outcomes	Cochrane’s Q test		MR-Egger intercept test		MR-PRESSO Global test *P-*value
			Q-value	*P*_Q_	Intercept	*P*_intercept_	
Significant estimates***	ALM	TH of lateral occipital	557.9186	0.0014	0.0001	0.2502	0.0013
		TH of pars opercularis	521.8636	0.0279	< 0.0001	0.6243	0.0263
Nominal significant Estimates*	ALM	SA of entorhinal	591.3911	< 0.0001	-0.0977	0.1677	< 0.0001
		SA of inferior parietal	566.5949	0.0005	0.5623	0.2294	0.0001
		SA of insula	725.5978	< 0.0001	0.3219	0.1268	< 0.0001
		SA of lateral occipital	676.8404	< 0.0001	0.5382	0.2324	< 0.0001
		SA of lateral orbitofrontal	707.1873	< 0.0001	-0.0776	0.7293	< 0.0001
		SA of lingual	640.5156	< 0.0001	0.3212	0.3621	< 0.0001
		SA of pars opercularis	582.9190	0.0001	0.1125	0.5641	< 0.0001
		SA of rostral middle frontal	630.7454	< 0.0001	0.2463	0.5948	< 0.0001
		TH of caudal middle frontal	548.4464	0.0034	-0.0001	0.1812	0.0049
		TH of cuneus	562.7317	0.0009	0.0001	0.3344	0.0007
		TH of entorhinal	579.0333	0.0001	0.0002	0.6215	0.0001
		TH of inferior parietal	565.2995	0.0006	0.0001	0.2516	0.0003
		TH of inferior temporal	497.9395	0.12	< 0.0001	0.9987	0.1171
		TH of lateral orbito frontal	633.0713	< 0.0001	0.0002	0.2157	< 0.0001
		TH of rostral middle frontal	499.4226	0.111	< 0.0001	0.8726	0.1141
		TH of superior temporal	552.3632	0.002	-0.0001	0.3445	0.0021
	BFP	TH of superior parietal	333.2079	0.0116	0.0002	0.3578	0.0116
		SA of lateral occipital	340.7171	0.0054	1.3823	0.1172	0.0057
		SA of pars opercularis	345.4539	0.0032	-0.1462	0.7246	0.0034
		SA of precentral	350.8345	0.0017	0.3289	0.6793	0.0026
		TH of caudal middle frontal	298.4377	0.1797	0.0001	0.4928	0.1801
		TH of fusiform	325.0243	0.0249	-0.0001	0.6511	0.0235
		TH of inferior parietal	266.5316	0.6635	< 0.0001	0.8473	0.6733
		TH of middle temporal	284.2107	0.3699	0.0004	0.1240	0.3730
		TH of precuneus	308.4761	0.0938	0.0001	0.7672	0.0950
	HGS-R	Global SA	298.4524	< 0.0001	24.3624	0.7255	< 0.0001
		TH of insula	221.0138	< 0.0001	0.0004	0.4126	< 0.0001
		TH of superior parietal	216.1541	< 0.0001	0.0002	0.5246	< 0.0001
		TH of superior temporal	158.2525	0.0273	0.0001	0.7649	0.0282
	HGS-L	SA of superior temporal	153.1053	0.0085	0.1955	0.8301	0.0079
		SA of temporal pole	119.6927	0.3391	-0.0567	0.7269	0.3439
		TH of bankssts	132.2531	0.1164	< 0.0001	0.9217	0.1261
		TH of superior temporal	154.7636	0.0067	0.0001	0.8901	0.0062

***Significant estimate is defined as IVW-derived *P* < 1.74*10^− 4^, *nominal significant estimate is defined as IVW-derived *P* < 0.05. Cochran’s Q-derived *P*-value, MR-Egger intercept-derived *P*-value and MR-PRESSO Global test *P* < 0.05 is significant

**Table 5:** Heterogeneity and Pleiotropy tests of the causal effects of brain structure on sarcopenia-related traits (Inverse Mendelian randomization Analysis)

	Exposures	Outcomes	Cochrane’s Q test		MR-Egger intercept test		MR-PRESSO
			Q-value	*P*_Q_	Intercept	*P*_intercept_	Global test *P*-value
**Significant**	TH of bankssts	ALM	4.7903	0.9409	-0.0139	0.0739	0.9507
estimates***	TH of frontal pole	ALM	33.9824	0.6978	0.0027	0.3071	0.7871
	TH of rostral anterior cingulate	ALM	150.4143	0.9993	0.0027	0.2841	0.9999
	TH of temporal pole	ALM	14.7525	0.1941	-0.0046	0.1105	0.325
	TH of temporal pole	HGS-R	70.4608	1	0.0008	0.1632	0.998
	TH of pars triangularis	HGS-L	92.0503	0.1683	0.0011	0.0992	0.2038
**Nominal significant**	TH of cuneus	ALM	4.3253	0.9991	0.0017	0.3984	0.9989
estimates*	TH of fusiform	ALM	11.1867	0.972	-0.0002	0.9237	0.9753
	TH of inferior parietal	ALM	7.2798	0.2006	-0.0047	0.0956	0.2798
	SA of middle temporal	ALM	44.1866	0.4638	-0.0002	0.8741	< 0.0001
	TH of pars orbitalis	HGS-R	96.5648	0.9957	0.0006	0.2637	0.9952
	TH of pars triangularis	BFP	43.7956	0.9997	0.0002	0.7372	0.9997

***Significant estimate is defined as IVW-derived *P* < 1.74*10^− 4^, *nominal significant estimate is defined as IVW-derived *P* < 0.05. Cochran’s Q-derived *P*-value, MR-Egger intercept-derived *P*-value and MR-PRESSO Global test *P* < 0.05 is significant

### Forward Mendelian randomization

At the global level, the genetically predicted ALM was significantly associated with increased cortical SA (β = 2925.4100 mm^2^, 95% CI: 2162.5185 mm^2^ to 3688.2924 mm^2^, *P* < 0.0001) but had no relationship with TH (β = -0.0010 mm, SE = 0.0022, *P* = 0.6437). However, because the *P*-value for the MR-Egger intercept is < 0.05, this data was excluded. At the functional region level, ALM was found to significantly decrease the TH of lateral occipital gyrus (β = -0.0079 mm, 95% CI: -0.0117 mm to -0.0041 mm, *P* < 0.0001) and notably increase the TH of pars opercularis gyrus (β = 0.0080 mm, 95% CI: 0.0042 mm to 0.0117 mm, *P* < 0.0001). Heterogeneity was observed with a Cochran Q-derived *P-*value < 0.05. As the random-effects IVW was used as the main result, heterogeneity is acceptable [39]. The *P*-value for the MR-Egger intercept is > 0.05 as well. Moreover, the genetically predicted ALM was nominally associated with reduced SA of lateral occipital, lingual, and rostral middle frontal gyrus, and TH of cuneus, entorhinal, inferior parietal, inferior temporal, lateral orbitofrontal, superior parietal gyrus. ALM was also nominally associated with increased SA of entorhinal, inferior parietal, insula, lateral orbitofrontal, pars opercularis, and supramarginal gyrus, and TH of caudal middle frontal, insula, rostral middle frontal, and superior temporal gyrus. Cochran’s Q statistic indicated that heterogeneity was only not detected regarding the TH of the inferior temporal (*P*_Q_ = 0.1200) and rostral middle frontal gyrus (*P*_Q_ = 0.1110) (Table 4).

Meanwhile, BFP was nominally associated with decreased SA of the precentral gyrus, and TH of superior parietal gyrus, fusiform, middle temporal, and superior temporal gyrus. And genetically predicted BFP was nominally associated with increased SA of lateral occipital, and pars opercularis gyrus, and TH of caudal middle frontal, inferior parietal, and precuneus gyrus. There was no heterogeneity in the TH of caudal middle frontal, inferior parietal, middle temporal, precuneus, and superior temporal gyrus. Horizontal pleiotropy existed in the TH of the superior temporal gyrus (Table 4).

HGS-R showed nominal association with increased global SA, TH of the insula and superior temporal gyrus and decreased TH of the superior parietal gyrus. Additionally, HGS-L was nominally associated with raised SA of superior temporal and temporal pole gyrus, and TH of bankssts and superior temporal gyrus. Heterogeneity and pleiotropy tests are presented in Table 4.

### Reverse Mendelian randomization

In the reverse MR analysis, we observed significant negative correlations between the TH of bankssts and ALM (β = -0.434 mm, 95% CI: -0.6514 mm to -0.2165 mm, *P* < 0.0001). The TH of frontal pole, rostral anterior cingulate, and temporal pole showed significant positive correlations with ALM (β = 0.2981 mm, 95% CI: 0.2412 mm to 0.355 mm, *P* < 0.0001; β = 0.2672 mm, 95% CI: 0.2324 mm to 0.302 mm, *P* < 0.0001; β = 0.3778 mm, 95% CI: 0.2478 mm to 0.5078 mm, *P* < 0.0001). The TH of temporal pole was significantly positively correlated with right-hand grip strength (HGSR) (β = 0.1596 mm, 95% CI: 0.1349 mm to 0.1843 mm, *P* < 0.0001). The TH of pars triangularis was significantly positively correlated with left-hand grip strength (HGSL) (β = 0.3251 mm, 95% CI: 0.2339 mm to 0.4163 mm, *P* < 0.0001). The TH of pars orbitalis was significantly negatively correlated with right-hand grip strength (HGSR) (β = -0.0848 mm, 95% CI: -0.129 mm to -0.0405 mm, *P* = 0.0002). TH of cuneus, TH of fusiform, and SA of middle temporal are nominally significantly positively associated with ALM. TH of inferior parietal is nominally significantly negatively associated with ALM. TH of pars orbitalis is nominally significantly negatively associated with HGSR, and TH of pars triangularis is nominally significantly positively associated with BFP. Detailed information is provided in Table 3.

Table 5 shows that the causal effects between exposure and outcome did not exhibit significant heterogeneity (*P* > 0.05), indicating good consistency of the results. The MR-Egger intercept test also did not show significant pleiotropy (*P*_intercept_ > 0.05), suggesting that the results were not significantly affected by horizontal pleiotropy. Additionally, the MR-PRESSO global test essentially indicates no horizontal pleiotropy.

## Discussion

**Fig. 2:**
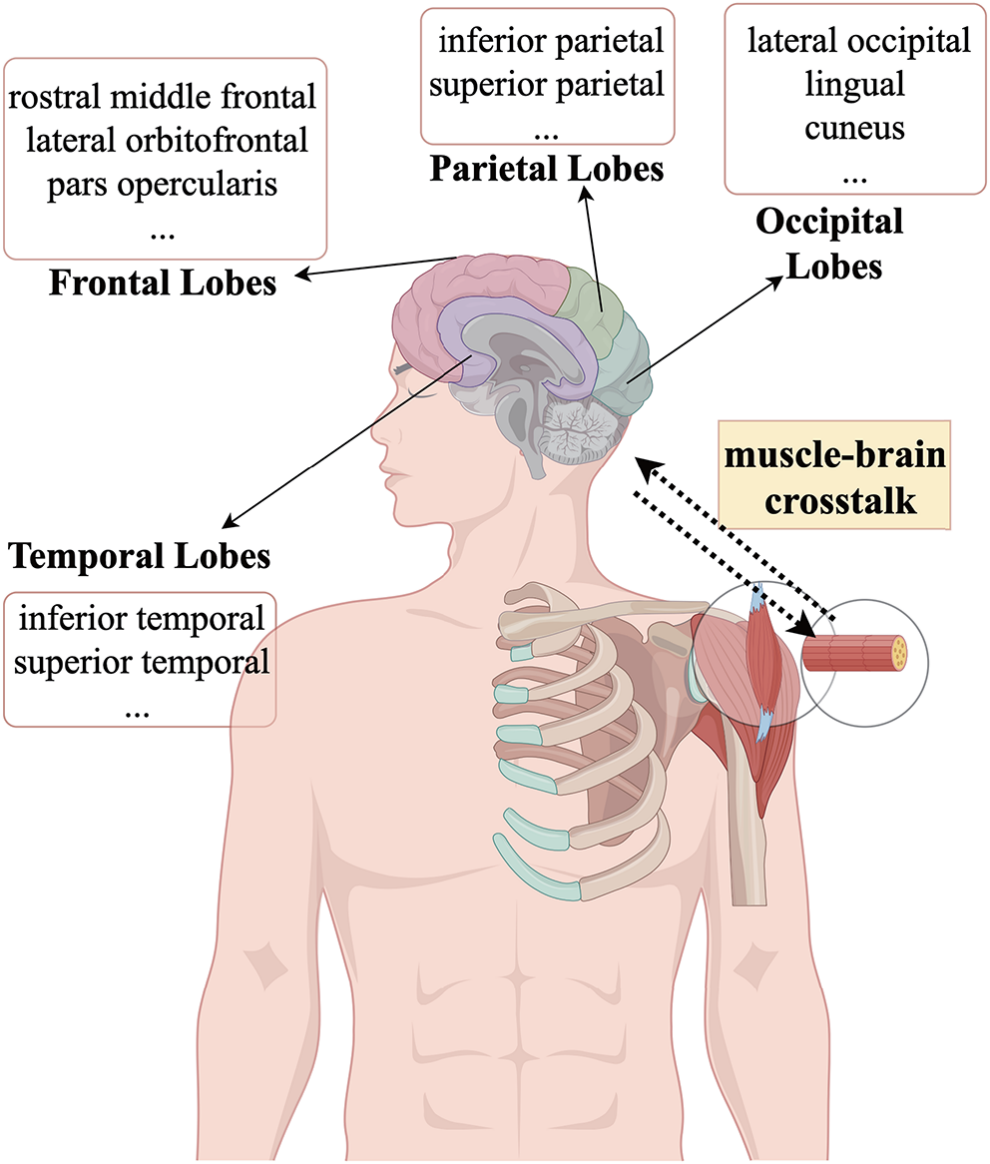
Muscle and cerebral cortex. Using a bidirectional two-sample Mendelian randomization framework, we have uncovered a causal relationship between sarcopenia-related traits and alterations in brain cortical structure, thereby supporting the existence of the muscle-brain axis (By Figdraw)

In this bidirectional two-sample Mendelian randomization study, we provided reliable evidence for the bidirectional causal relationships between sarcopenia-related traits and brain cortical structures. We observed that ALM, BFP, HGS-R, and HGS-L mutually influence the brain cortex. These findings imply a pathophysiological interplay between muscle degradation and brain functionality (Fig. 2), thus shedding light on the existence of a muscle-brain axis [15, 16].

The cerebral cortex is widely acknowledged as the primary hub for human cognition, encompassing a broad range of neuronal cell types that form the foundational structure for intricate functions [40]. Cognitive decline is characterized by a deterioration in one or more areas of cognitive ability, including speech, vision, hearing, memory, and motor skills [15]. Current research suggests that reductions in cortical thickness have been linked to diminished intelligence and cognitive decline [41, 42], and alterations in both regional and global cortical surface area and thickness are strongly correlated with various neuropsychiatric disorders [33]. Furthermore, there is also growing evidence linking sarcopenia to neurological disorders [8]. Therefore, the link between sarcopenia and brain structure deserves further exploration.

The primary finding of this study is the significant and positive correlation between ALM and the TH of the pars opercularis, a brain region known for its crucial role in language function [33]. Studies have found that patients with renal injury [43]and inflammatory bowel disease [44] also exhibit a decrease in the TH of the pars opercularis. Another significant finding was the observed negative correlation between ALM and the TH of the lateral occipital region. The lateral occipital region plays a vital role in visual processing, specifically in object recognition and spatial perception [45]. Furthermore, a decrease in the TH of the lateral occipital region was also evident among children with sensorineural hearing loss [46] and autism spectrum disorders [47]. In conjunction with other normally significant elements (*P*_ivw_ < 0.05), and alignment with previous observational studies, our findings suggest that ALM is not associated with total cortical area and volume. However, there are some correlations between ALM and certain prefrontal cortical regions responsible for motor function [20, 48], as well as the parietal lobe involved in attention and somatic senses [18, 49]. Overall, sarcopenia exerts an impact on language and visual functions by influencing specific brain areas. Further research is required to elucidate the underlying mechanisms at play.

BFP shows a significant positive correlation with the TH of the superior parietal region, which is implicated in perceptual functions such as spatial localization [50]. This is consistent with previous studies showing an association between body mass index or body fat and brain structure [51], as well as a negative correlation between body fat percentage and cortical thickness [52]. HGS-R is significantly and positively correlated with total cerebral cortical surface area. Greater grip strength is linked to larger gray matter areas [53, 54], at the same time, the normally significant elements (*P*_ivw_ < 0.05) should also be given attention, especially the temporal lobe region, which is associated with hearing, memory, emotion, and some aspects of language [53, 55].

Muscle mass or strength affects certain brain regions, explaining to some extent the cognitive decline in patients with sarcopenia, which also somewhat supports the theory of muscle-brain crosstalk. The link between sarcopenia-related traits and cognitive decline in the elderly has also been explained by the role of myokines in regulating muscle-brain crosstalk [56]. Skeletal muscle secretes myokines, such as brain-derived neurotrophic factor (BDNF), during contraction [57, 58]. A univariate analysis has verified the significantly lower levels of BDNF in patients with severe sarcopenia [59]. BDNF involves a variety of cortical regulatory functions and promotes the growth of neurons and the formation of synapses in nerve cells [60]. Besides, sarcopenia reduces the level of regulating myokines that play a crucial role in the muscle-brain cross-talk [61], including interleukin 6 (IL-6) [62], insulin-like growth factor1 (IGF-1) [63], etc. In vitro studies showed that IL-6 serves as an important regulator of neuron function in health and disease [64]. Through exercise can increase secretory myokines [56], can improve brain structure [65], and enhance cognition [66].

We have also discovered that part of the muscle mass degeneration may be due to abnormalities in brain structure. Reverse MR analysis suggests that the cortical thickness of certain brain regions may play a significant role in maintaining muscle mass and function. The frontal pole, a key area for higher cognitive functions, is closely associated with the weakening of motivation for exercise and a decrease in physical activity [67]. The TH of temporal pole shows a significant correlation with ALM and HGS-R, potentially regulating muscle status indirectly by influencing behavioral patterns such as exercise habits or social activities [68, 69]. Other areas, such as the TH of rostral anterior cingulate, involved in emotional regulation and control of the autonomic nervous system, indirectly affect physical activity [70], thus regulating muscle mass and function. There is a correlation between changes in brain structure and muscle mass [71, 72], and research now indicates that brain CT scans can be used to predict muscle status [3]. Brain regions can adjust according to their respective functions, thereby influencing movement and affecting muscle mass and function [73]. In summary, this study underscores the reciprocal relationship between sarcopenia-related characteristics and brain health. It highlights the importance of paying attention to brain health in patients with sarcopenia and suggests that regular exercise can enhance both muscle mass and cognitive function [74]. Furthermore, future research should further investigate the specific mechanisms linking sarcopenia and brain health to develop more effective intervention strategies and improve patients’ quality of life.

The major strength of this study lies in its MR design [75], which provides a high level of evidence to minimize bias and prevent reverse causal associations. In our study, we selected robust genetic instruments as instrumental variables with F-statistics greater than 10. We also eliminated SNPs associated with confounding factors such as smoking and hyperlipidemia to minimize the impact of confounding on the results. The study performed an extensive analysis of GWAS data, assessing two measures of total cortical surface areas and thicknesses, 34 specific brain regions, and four different sarcopenia exposure profiles. To ensure the accuracy and validity of the results, various MR analysis methods were employed, including sensitivity analyses, which bolstered the reliability of the MR causal analysis. Conducting studies on brain structures often involves challenges such as the requirement for large sample cohorts and follow-up imaging assessments. However, MR studies overcome these limitations by allowing for a prompt evaluation of the causal relationship between the exposure and the outcome.

On the other hand, there are some limitations to consider. Firstly, the data utilized in this study predominantly originated from European populations and may not be representative of other populations. Secondly, although the study reported changes in cortical structure in patients with sarcopenia, further investigations are required to elucidate the underlying mechanisms. Lastly, residual bias cannot be eliminated even when multiple validity tests and MR-PRESSO procedures are utilized to control for confounding due to the recognized limitation of MR techniques.

## Conclusion

This study conducted a comprehensive bidirectional MR analysis of the relationship between ALM, BFP, HGS-R, HGS-L, and cortical structures. The results indicate that the reduction in muscle mass is not only a consequence of changes in cortical alterations but also affects the structure of the brain cortex, with the underlying mechanisms requiring further investigation. Our findings further support the muscle-brain axis theory and link sarcopenia with neurocognitive diseases, providing new strategies for the prevention and intervention of sarcopenia and cognitive decline.

## Electronic supplementary material

Below is the link to the electronic supplementary material.

Supplementary Material 1

## Data Availability

No datasets were generated or analysed during the current study.
