# Supplementary material for: Causal association of sarcopenia-related traits with brain cortical structure: a bidirectional Mendelian randomization study
Source: Aging Clin Exp Res. 2025 Feb 27;37(1):57. doi: 10.1007/s40520-025-02977-x (PMC11868162; doi:10.1007/s40520-025-02977-x)
Supplement: Supplementary file 1 — Supplementary Material 1 [file 40520_2025_2977_MOESM1_ESM.pdf]

Table S1 Descriptions of study cohorts participating in Grasby's study

Cohort	Study Design	Ancestry	Total N	Females	Mean Age	SD	Min Age	Max Age	Healthy N
1000BRAINS	Population-based	European	775	346	67.3	6.7	53.4	85.4	775
ADNI1	Case-control (AD, MCI, healthy control)	European	735	299	74.8	6.8	54.0	90.0	204
ADNI2GO	Case-control (AD, MCI, healthy control)	European	649	297	72.4	7.1	55.0	91.4	564
ALSPAC[a]	Population-based	European	391	0	19.6	0.9	18.0	21.5	391
ASRB	case-control	European	233	138	38.5	11.3	19.0	64.0	75
BETULA	Population-based	European	311	169	62.4	13.3	25.5	81.3	311
BIG-Affy	Population-based	European	1180	688	22.6	3.8	18.0	40.0	1180
BIG-PsychChip	Population-based	European	432	206	22.5	4.4	17.0	44.0	432
BONN	Population-based	European	102	0	38.2	6.6	29.0	50.0	102
BrainScale	Population-based Twin Study	European	242	131	10.0	1.3	9.0	15.0	242
CARDIFF	Population-based	European	270	194	24.8	6.9	18.0	58.0	270
DNS-V3	Population-based	European	324	168	19.7	1.2	18.0	22.0	324
DNS-V4	Population-based	European	191	108	19.9	1.2	18.0	22.0	191
EPIGEN	Epilepsy cases	European	178	104	38.4	13.2	14.0	85.0	0
FOR2107	Population-based plus Affective disorders cases	European	785	474	34.4	13.0	18.0	65.0	416
GIG	Population-based	European	283	168	24.2	2.4	19.0	31.0	283
GSP	Population-based	European	442	251	21.4	3.2	18.0	35.0	442
HUBIN	Case-control (SCZ, healthy controls)	European	177	55	41.9	8.2	19.4	56.3	97
HUNT	Population-based	European	876	462	58.9	4.2	50.5	66.8	876
IMAGEN	Population-based	European	1358	725	14.6	0.4	12.9	17.2	1358
IMpACT	Case-control (ADHD, healthy controls)	European	238	140	40.8	12.0	20.0	70.0	113
LBC1936	Population-based	European	604	285	72.7	0.7	71.0	74.2	604
LIBD	Case-control (SCZ, healthy controls, unaffected siblings)	European	484	214	33.2	10.1	18.6	61.6	310
MCIC	Case-control (SCZ, healthy controls)	European	162	56	33.7	11.2	18.0	59.0	94
MooDS	Population-based	European	282	129	33.6	9.8	18.0	51.0	282
MPIP	Case-control (MDD, healthy controls)	European	550	318	48.3	13.3	18.0	87.0	177
MPRC	Case-control (SCZ, healthy controls)	European	387	205	37.2	14.6	10.0	79.0	214
MÜNSTER	Case-control (MDD, healthy controls)	European	985	561	35.8	12.1	17.0	65.0	741
NCNG	Population-based	European	321	218	51.6	16.7	19.4	82.3	321
NESDA	Case-control (depression, anxiety, healthy controls)	European	254	171	37.5	10.2	18.0	57.0	55

NeuroIMAGE	Case-control (ADHD, healthy controls)	European	210	68	17.1	3.2	8.2	25.0	61
NTR	Population-based Twin Study	European	322	197	29.4	11.0	12.0	56.0	322
OATS	Population-based Twin Study	European	360	237	70.5	5.1	65.0	89.0	360
PAFIP	Case-control (SCZ, healthy controls)	European	112	42	28.3	8.0	16.1	50.7	14
PDNZ	Case-control (Parkinson's disease, healthy controls)	European	164	56	68.2	7.8	45.5	81.9	47
PING	Population-based	European	337	151	11.8	4.7	3.4	20.8	337
PPMI	Case-control	European	414	137	61.7	9.6	30.6	84.9	124
QTIM	Population-based Twin Study	European	996	645	22.4	3.3	15.4	30.1	996
SHIP	Population-based	European	1118	579	55.8	12.8	30	90	1118
SHIP-Trend	Population-based	European	891	499	50.4	13.5	22	81	891
Sydney MAS	Population-based	European	494	274	78.4	4.7	70.5	90.1	494
SYS	Family-based study	European	1675	884	28.3	17.4	11	65.4	1675
TCD-NUIG	Case-control (healthy control, schizophrenia)	European	192	108	29.9	10.4	18	63	156
TOP	Case-control (SCZ, BD, other psychoses, healthy controls)	European	505	253	35.2	10.2	18.2	64.8	216
TOP3T	Case-control (SCZ, BD, other psychoses, healthy controls)	European	400	181	33.2	11.9	18	78	296
UiO2016	Case-control	European	229	98	31.8	10.0	15.6	60.5	90
UiO2017	Case-control	European	308	173	42.1	17.9	13.1	89.0	247
UKBB	Population-based	European	10083	5261	62.8	7.4	46.4	46.4	10083
UMCU	Case-control / family study (SCZ, BD, offspring, healthy controls)	European	698	373	33.1	14.1	9	67	548

This table is cited from Grasby KL, Jahanshad N, Painter JN, Colodro-Conde L, Bralten J, Hibar DP, et al. The genetic architecture of the human cerebral cortex. Science. 2020;367(6484).

Figure S1 Scatter plots and leave-one-out plots of significant estimates in both forward and reverse Mendelian randomization analyses. (A) ALM on TH of lateral occipital; (B) ALM on TH of pars opercularis; (C) TH of bankssts on ALM; (D) TH of frontal pole on ALM; (E) TH of rostral anterior cingulate on ALM; (F) TH of temporal pole on ALM; (G) TH of temporal pole on HGS-R; (H) TH of pars triangularis on HGS-L.

A

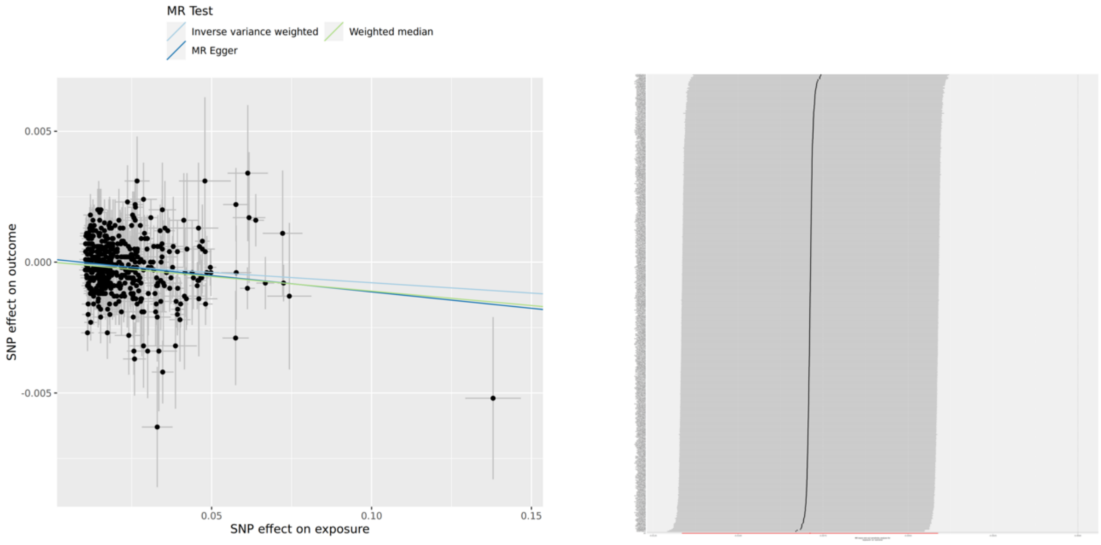

B

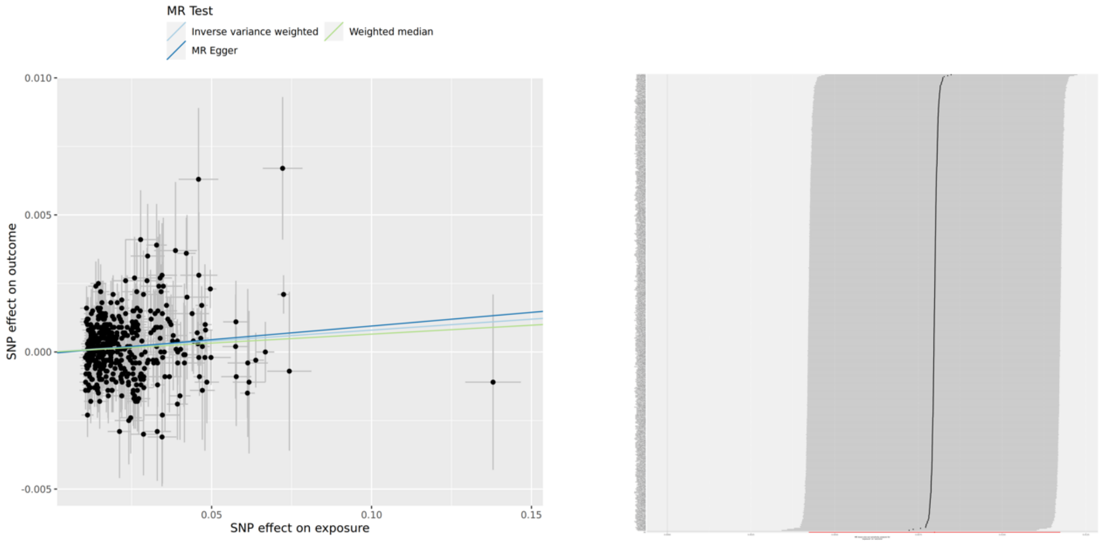

C

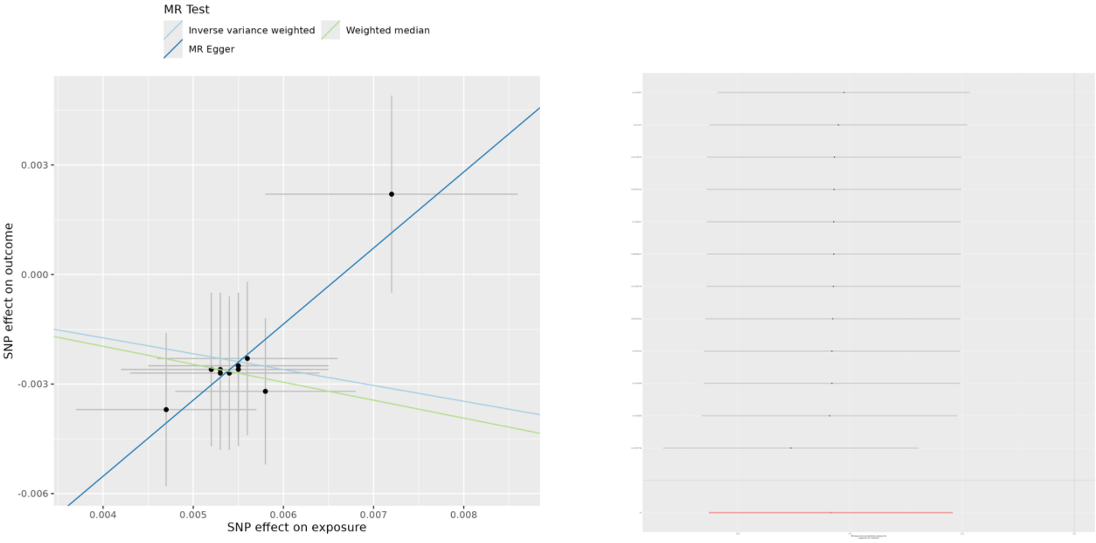
MR Test
Inverse variance weighted
Weighted median
MR Egger
0.003
0.000
-0.003
-0.006
SNP effect on outcome
0.004
0.005
0.006
0.007
0.008
SNP effect on exposure

D

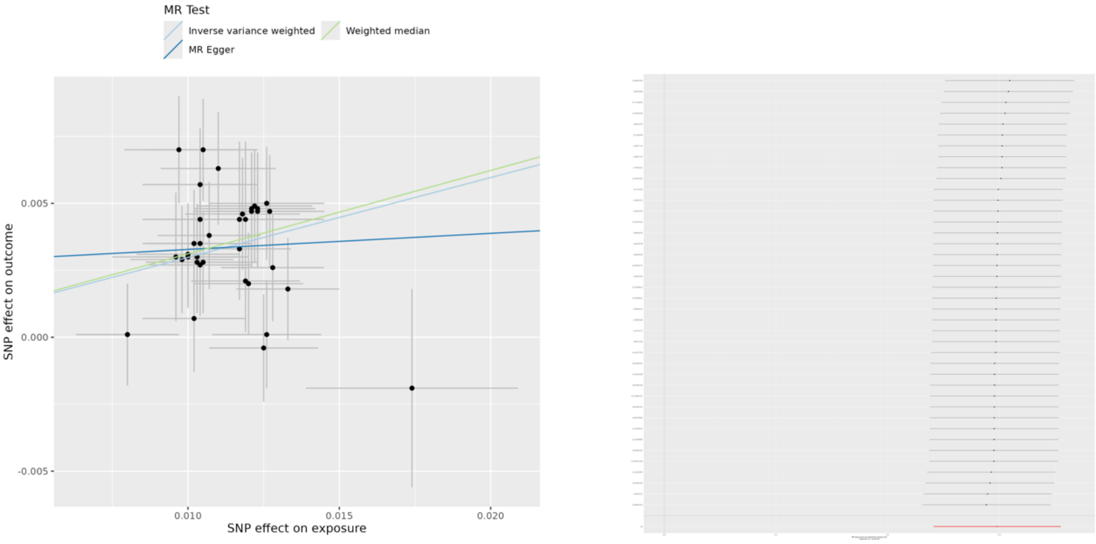
MR Test
Inverse variance weighted
Weighted median
MR Egger
0.005
0.000
-0.005
SNP effect on outcome
0.010
0.015
0.020
SNP effect on exposure

E

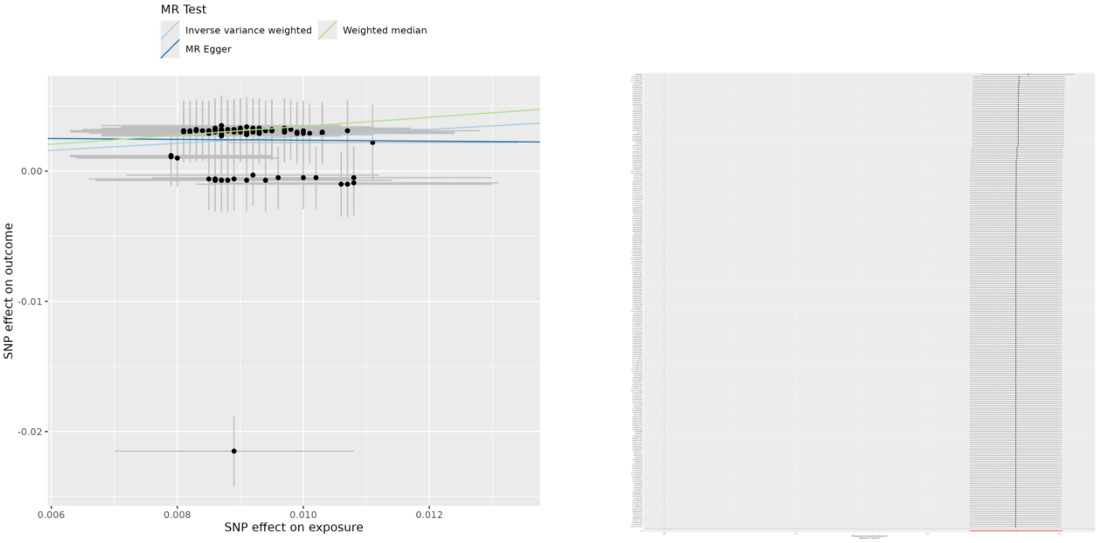

MR Test
Inverse variance weighted
Weighted median
MR Egger
SNP effect on outcome
0.00
-0.01
-0.02
0.006
0.008
0.010
0.012
SNP effect on exposure

F

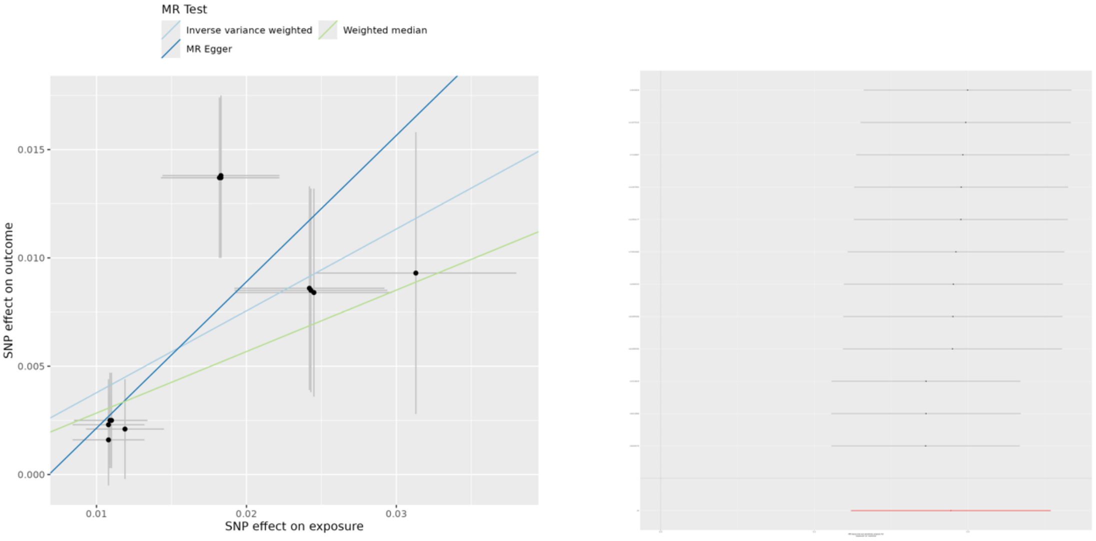

MR Test
Inverse variance weighted
Weighted median
MR Egger
SNP effect on outcome
0.015
0.010
0.005
0.000
0.01
0.02
0.03
SNP effect on exposure

G

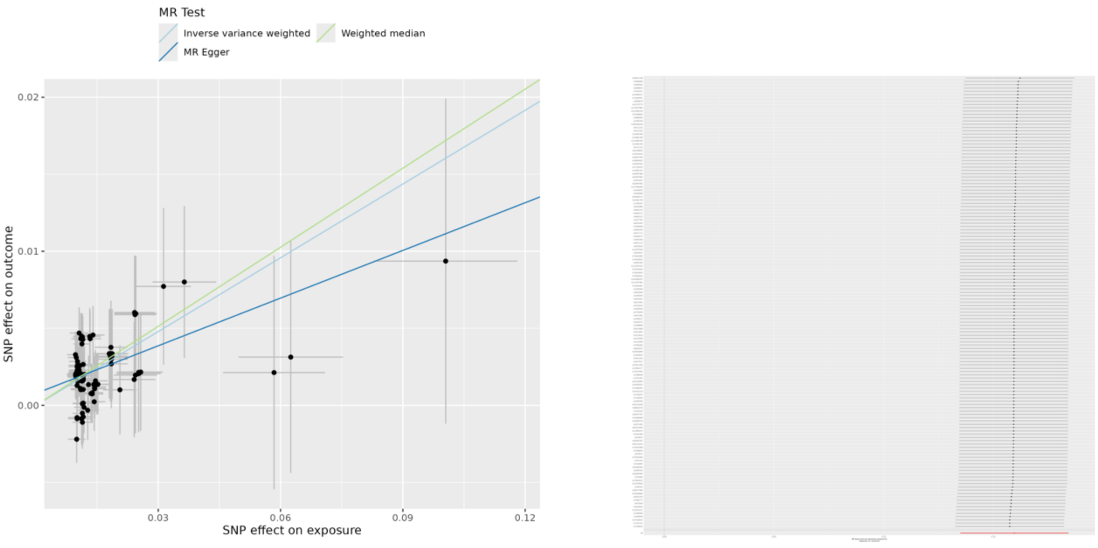
MR Test
Inverse variance weighted
Weighted median
MR Egger
SNP effect on outcome
SNP effect on exposure
0.02
0.01
0.00
0.03
0.06
0.09
0.12

H

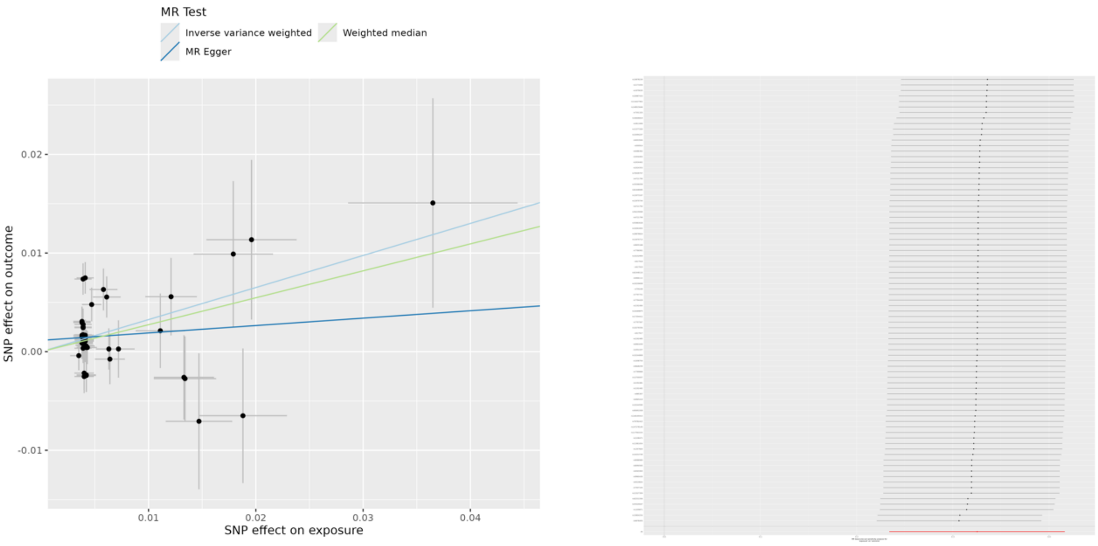
MR Test
Inverse variance weighted
Weighted median
MR Egger
SNP effect on outcome
SNP effect on exposure
0.02
0.01
0.00
-0.01
0.01
0.02
0.03
0.04
